# Effect of DiD Carbocyanine Dye Labeling on Immunoregulatory Function and Differentiation of Mice Mesenchymal Stem Cells

**DOI:** 10.1155/2014/457614

**Published:** 2014-12-11

**Authors:** Maryam Sadat Mohtasebi, Fatemeh Nasri, Eskandar Kamali Sarvestani

**Affiliations:** ^1^Department of Immunology, Shiraz University of Medical Sciences, Shiraz 7134845794, Iran; ^2^Student Research Committee, Shiraz University of Medical Sciences, Shiraz 7134845794, Iran; ^3^Autoimmune Diseases Research Center, Shiraz University of Medical Sciences, Shiraz 7134845794, Iran

## Abstract

Mesenchymal stem cells (MSCs) have been used to treat a variety of degenerative disorders. Labeling of MSCs with an appropriate tracer is vital to demonstrate the *in vivo* engraftment and differentiation of transplanted MSCs. DiD is a lipophilic fluorescent dye with near infrared emission spectra that makes it suitable for *in vivo* tracing. Therefore, in the present study the consequences of DiD labeling on induction of oxidative stress and apoptosis as well as inhibition of biological functions of mesenchymal stem cells (MSCs) were investigated. DiD labeling did not provoke the production of ROS, induction of apoptosis, or inhibition of production of immunosuppressive factors (PGE2 and IL-10) by MSCs. In addition, there were no statistical differences between DiD-labeled and unlabeled MSCs in suppression of proliferation and cytokine production (IFN-*γ* and IL-17) by *in vitro* stimulated splenocytes or improvement of clinical score in EAE after *in vivo* administration. In addition, DiD labeling did not alter the differentiation capacity of MSCs. Taken together, DiD can be considered as a safe dye for *in vivo* tracking of MSCs.

## 1. Introduction

Among stem cells with the ability to differentiate into various cell types, mesenchymal stem cells (MSCs) with immunosuppressive functions are promising tools for therapeutic applications in autoimmune diseases even in allogeneic settings. The efficacy of MSCs in the treatment of autoimmune diseases is principally dependent on suppression of ongoing inflammatory responses by production of anti-inflammatory cytokines as well as their ability in differentiation into functional cells [1–3]. To investigate the migration of MSCs to damaged tissue or their* in vivo* differentiation into injured cells, availability of an appropriate tracing system is necessary. Different imaging methods have been used to track the location of injected MSCs without sacrificing the animal. However, noninvasive imaging techniques based on bioluminescence, single-photon emission computed tomography using enzymatic conversion/retention, and positron emission tomography need stable integration of the transgene [4, 5]. The requirement for genetic manipulation of stem cell and intravenous injection of potentially immunogenic substrate for each imaging session are among disadvantages of these methods. Nuclear imaging has also several disadvantages including exposure to radiation, high costs, and short half-life of almost all of nuclear tracers [6]. The technique of choice to trace the cell fates after injection is fluorescence-based* in vivo* optical imaging that is a safe and noninvasive method with capability of* in vivo* chasing of labeled cells over time. Beside biocompatibility and resolution concerns, the lack of influence of selected tracer on desired biological functions of MSCs is also quite important. Therefore, any tracer that is used for* in vivo* MSCs-tracking should affect neither immunomodulatory functions nor differentiation capacity of MSCs.

Fluorescent lipophilic carbocyanine dyes are insoluble in water, but their fluorescence is readily detected when incorporated into membranes. They are classified as one of the most appropriate families of dyes in labeling and tracking. Lipophilic carbocyanine dyes incorporate into cell membranes and diffuse laterally within the cellular plasma membranes, resulting in staining of the entire cell [7, 8]. Simple staining procedure, structural similarity with cell membrane phospholipids, and prolonged dye retention in cells are among the advantages of these dyes for using in live organisms [9, 10]. Amongst the members of this family, DiD with long wavelength excitation and emission spectra is proper for* in vivo* imaging due to the fact that NIR fluorescence avoids the interference of the target tissue with the background fluorescence and creates a high contrast when the dye is tracked by* in vivo* imaging system [10, 11]. Interestingly, DiD has been used for labeling of MSCs with no interference with MSCs differentiation into chondrocytes [11]. However, the effects of DiD labeling on immunosuppressive functions and differentiation of MSCs towards an adipogenic or osteogenic cell fate remained to be investigated. Accordingly, the present study aimed to shed light on possible effects of DiD labeling on* in vitro* differentiation of DiD-labeled MSCs into adipogenic, osteogenic, and neural progenitor cells as well as* in vitro* and* in vivo* immunosuppressive function of DiD-labeled MSCs.

## 2. Materials and Methods

### 2.1. Animals

Female C57BL/6 and male Balb/C mice were purchased from Pasteur Institute of Iran and maintained under the 12 h light/dark condition at 25°C. Balb/C mice between 4 and 5 weeks of age were used for isolation of the MSCs. Experimental autoimmune encephalomyelitis (EAE) was induced in C57BL/6 mice. All the animals were maintained under National Institute of Health Guide for Care and Use of Laboratory Animals (NIH publication, 1985) in animal house of Shiraz University of Medical Sciences.

### 2.2. MSCs Isolation, Culture, Characterization, and DiD Labeling

MSCs were isolated from femur and tibia according to the previously reported method by Da Silva Meirelles and Nardi [12]. Briefly, bone marrow cells from femur and tibia of Balb/C mice were suspended in proper media and maintained in 5% CO_2_ atmosphere at 37°C with 95% humidity. After 24 h, the suspended cells were removed by changing the medium. After reaching 70–90% confluence, the adherent cells were trypsinized and moved to a new flask with suitable density. In order to separate the pure MSCs population, trypsinization of MSCs was repeated for at least 4 times when the cells reached suitable confluence. MSCs in passage 5 were trypsinized and analyzed with flow cytometry (BD FACSCalibur, USA) for surface markers using phycoerythrin- (PE-) conjugated anti-CD45, anti-CD44, and anti-Sca-1 as well as fluorescein isothiocyanate- (FITC-) conjugated anti-CD34 (eBioscience, UK) and anti-MHCII (BD Pharmingen, USA) antibodies. Proper isotype controls were used in all experiments. The data were collected and analyzed by Cell Quest and Flow Jo software (version 7.6), respectively. MSCs were labeled with different concentrations of DiD to determine the highest nontoxic concentration of the dye which could be traced after multiple division of MSCs. Accordingly, staining of MSCs was performed with 5 *μ*M of DiD. Briefly, 1 × 10^6^ MSCs were suspended in 1 mL DMEM containing 5 *μ*M DiD (AAT Bioquest, USA) for 20 min at 37°C, 95% humidity, and 5% CO_2_. The labeled cells were washed three times with PBS to remove excess DiD and used in further experiments.

### 2.3. Proliferation Capacity of DiD-Labeled MSCs

Proliferative capacity of the MSCs was assessed after labeling with 5 *μ*M DiD. A total of 2 × 10^3^ labeled and control cells were counted and plated in a 96-well plate. After a culture period of 30 h, the MSCs were pulsed with 0.5 *μ*Ci of [^3^H]-methylthymidine (MP Biomedical, USA) for 18 hours to determine the fold of expansion.

### 2.4. Reactive Oxygen Species (ROS) Production of MSCs

Production of ROS in the DiD-labeled MSCs was measured using dichlorofluorescein diacetate (DCFDA, Invitrogen, USA) staining [13]. DiD-labeled and unlabeled MSCs (2 × 10^5^) were incubated in a 6-well plate with 2 mL DMEM for one day. Then, DCFDA (final concentration 10 mM) was added to the culture for 30 min in CO_2_ incubator. After that, cells were washed with PBS for three times and trypsinised and the dye intensity was quantified by flow cytometer.

### 2.5. Mitochondrial Membrane Potential (MMP) of MSCs

Decrease of MMP is an early indicator of cell apoptosis. According to the procedure reported by Chang et al. [14], 2 × 10^5^ DiD-labeled or unlabeled MSCs were seeded in a 6-well plate and incubated with 40 nM of 3,3-dihexyloxacarbocyanine DiOC6(3) (Enzo life Sciences, USA) at 37°C for 20 min. After incubation, MSCs were washed and resuspended in PBS and their fluorescent intensity was determined by flow cytometry.

### 2.6. Differentiation of DiD-Labeled MSCs

MSCs cells were incubated with 5 *μ*M DiD for 20 min and kept in the culture for a day before starting the differentiation protocols. Control wells were treated with the same except for labeling with DiD. For differentiation towards adipocytes, ~70% confluent cells were cultured for 10 days in DMEM supplemented with 1 *μ*M dexamethasone, 0.5 *μ*M ascorbic phosphate, and 200 *μ*M indomethacin [15]. The medium was changed every 2-3 days. Intracellular accumulation of lipid vacuoles was also shown by staining with 0.5% Oil Red O for 10 minutes. Osteogenic differentiation was done with a medium supplemented with 1 *μ*M dexamethasone, 10 mM *β*-glycerophosphate, and 0.5 *μ*M ascorbic phosphate for 10 days [16]. Cells were fed by changing media approximately once every three days. Calcium deposition was revealed by 1% Alizarin Red staining for 10 minutes. For neural precursor (NP) differentiation, 1 × 10^6^/mL MSCs from passages 7–10 were cultured in neurobasal differentiation media on agarose gel coated plates which were used for bolster neurosphere induction as described by Shiri et al. [17]. Neurobasal media were supplemented by 1% B27, 1% insulin-transferring-selenite, 2 mM L-glutamine, 1% penicillin/streptomycin, 10% FBS, 1 ng/mL b-FGF, and 1 *μ*M DHEA. All materials for differentiation were purchased from Sigma, USA. After 7 days, neurosphere formation was identified by microscopic examination and the cells were collected to investigate the expression of neuronal stem cell marker (nestin) by real time-PCR. Total cellular RNA was isolated from neurosphere by RNAX plus kit (Cinnagen Company, Iran). In addition, cDNA was synthesized from 2 *μ*g total RNA using random hexamer primer and M-MuLV reverse transcriptase (Fermentas, USA) for 60 min at 42°C. Nestin primer sequences (forward, reverse) were as follows: 5′-TACAGAGTCAGATCGCTCAGATCC-3′, 5′-CAGCAGAGTCCTGTATGTAGCCAC-3′. Both primers had an intron spanning to avoid genomic DNA contamination. Moreover, PCR reactions were normalized using selective forward 5′-AGCTTCTTTGCAGCTCCTTCG-3′ and reverse 5′-CATCCATGGCGAACTGGTG-3′ primers for *β*-actin as internal control. The cDNA was amplified as follows: incubation at 95°C for 10 min followed by 40 cycles of 10 s at 95°C, 1 min at 60°C. The melting curves of the real-time PCR products were observed for specificity of the products after the end of the reaction.

### 2.7. Mixed Leukocyte Reaction (MLR)

MLR in the presence of MSCs was done to compare the immunomodulatory function of unlabeled and DiD-labeled MSCs. 3 × 10^4^ cells from passages 4–6 were seeded in 96-well plates in DMEM containing 10% FBS, 100 U/mL penicillin, and 100 *μ*g/mL streptomycin. After 8 hours, the MSCs were washed twice with PBS and 3 × 10^5^ CFSE-labeled splenocytes (1.5 × 10^5^ from C57BL6 and 1.5 × 10^5^ from BALB/c mice) were added to the MSCs. Splenocytes were labeled with 10 *μ*M CFSE in 150 *μ*L of RPMI for 15 minutes. 1 *μ*g/ml Concanavalin A was added to each well except unstimulated splenocytes (negative control). After five days, the splenocytes were harvested and the rate of proliferation was evaluated based on diminution of CFSE fluorescent intensity in cells using flow cytometry.

### 2.8. Treatment of EAE by DiD-Labeled MSCs

MOG_35–55_ peptide (MEVGWYRSPFSRVVHLYRNGK, Peptron, Korea) was used to induce chronic EAE in C57BL/6 mice [18]. Briefly, 200 *μ*g of MOG_35–55_ diluted in 200 *μ*L PBS and emulsified with 200 *μ*L incomplete Freund's adjuvant (Gibco, Germany) and 1 mg/mL heat inactivated mycobacterium tuberculosis (Difco, USA) in PBS was injected subcutaneously. After MOG_35–55_ administration, 200 ng pertussis toxin (List Biological Laboratory, USA) was injected intraperitoneally on the day of immunization and two days later. Clinical scores of the EAE were recorded at least 3 times per week. EAE sign was scored as follows: 0 = no disease, 1 = limp tail, 2 = hind limb paralysis, 3 = paralysis of all four limbs, 4 = moribund condition, and 5 = death [19]. The mice were divided into three groups each containing 5 mice with the mean clinical score of 2.2. One million DiD-labeled or unlabeled MSCs in 0.2 mL of PBS were injected on days 22, 29, and 36 into the peritonea of the treated groups, while the control EAE mice received 0.2 mL of diluents on the same days. The mice were followed up until day 45. To examine the effect of DiD-labeled MSCs on antigen-specific immunosuppression, 24 days after initial administration of MSCs, splenocytes from EAE mice (treated and untreated) were stimulated with MOG_35–55_ peptide and proliferation was assessed by ^3^H-thymidine incorporation. Briefly, 2 × 10^5^ splenocytes from treated- and untreated-EAE mice were stimulated by 20 *μ*g/well of MOG_35–55_. After a culture period of 72 h, splenocytes were pulsed with 0.5 *μ*Ci/well of [^3^H]-methylthymidine (MP Biomedical, USA) for 18 h to determine the fold of expansion. Radionuclide uptake was measured by *β*-scintillation counter (Wallac, Germany).

### 2.9. Cytokine Detection by ELISA

Supernatants of 1 × 10^6^ DiD-labeled and unlabeled MSCs in 2 mLs DMEM were collected after 24 h of culture and kept frozen at −70°c until determination of cytokine levels. Supernatants of MLR were also collected and kept in the same condition for cytokine analysis. The levels of PGE2 and IL-10 in the supernatants of MSCs as well as levels of PGE2, IL-10, IFN-*γ*, and IL-17 in the supernatants of MLR were quantitatively analyzed by enzyme-linked immunosorbent assay (ELISA) according to the manufacturer's instructions (Mabtech, UK). Sera from treated- (with labeled and unlabeled MSCs) and untreated-EAE mice were also evaluated for the levels of inflammatory (IFN-*γ* and IL-17) and anti-inflammatory (IL-10) cytokines by ELISA.

### 2.10. Data Analysis

The data are presented as mean ± SD. Kruskal-Wallis test was used to check the overall difference between groups. Also Mann-Whitney *U* was used to analyze the difference in clinical scores and cytokine levels between treated- and untreated-EAE groups. SPSS 15 software was used for statistical analysis. *P* < 0.05 was considered as statistically significant.

## 3. Results

Both labeled and unlabeled MSCs grow in a homogenous fibroblast shaped pattern. In addition, flow cytometric analysis of MSCs for expression of CD44, Sca-1, MHC II, and hematopoietic stem cell markers (CD45 and CD34) showed more than 95% purity in the fifth passage (data not shown).

### 3.1. Proliferation Capacity of DiD-Labeled MSCs

**Figure 1:**
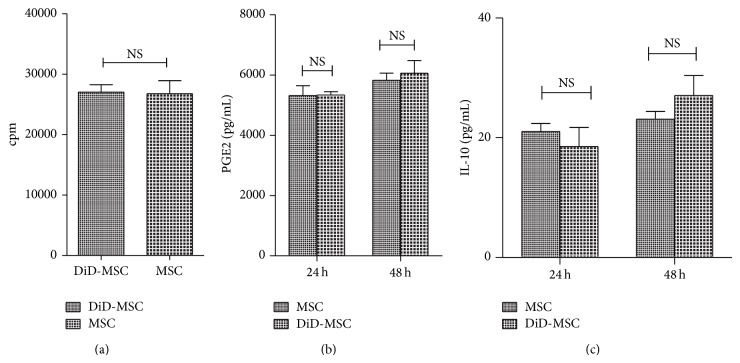
Evaluation of IL-10 and PGE2 production and proliferation assay in intact and DiD-labeled MSCs. No significant difference was observed between unlabeled and DiD-labeled MSCs in proliferation capacity by [^3^H]-thymidine incorporation (a), levels of PGE2 (b), and IL-10 (c) production after 24 h and 48 h in culture. These data were expressed as mean ± SD of three independent experiments in triplicate.

To assess possible effect of DiD labeling on proliferation of MSCs, the cells were stained with DiD and after 48 hours of culture were pulsed with [^3^H]-methylthymidine for 18 hours. No significant differences were observed between DiD-labeled and intact MSCs (27014 ± 2001 CPM and 26747 ± 3540 CPM, resp.; *P* = 0.79; Figure 1(a)).

### 3.2. Multilineage Differentiation Capacity of DiD-Labeled MSCs

**Figure 2:**
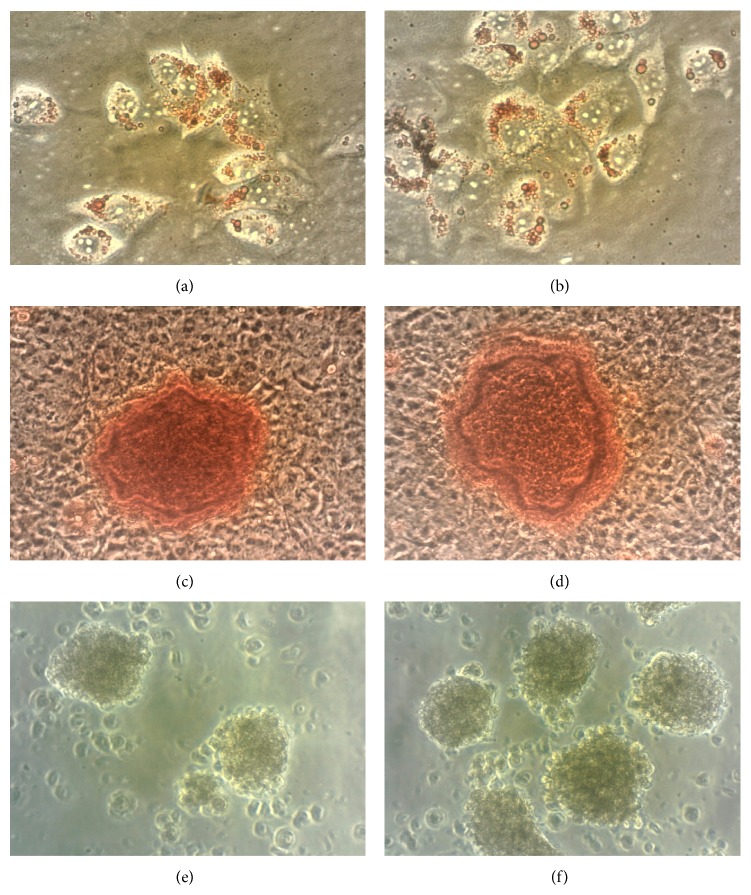
Lineage differentiation of untagged (a, c, and e) and DiD-labeled MSCs (b, d, and f) into adipocytes, osteocytes, and NPs. Culturing of unlabeled and DiD-labeled MSCs in differentiation media followed by staining with Oil Red O for lipid (a and b, resp.; original magnifications: 400x) or Alizarin Red for calcium deposition (c and d, resp.; original magnifications: 400x) and microscopic examinations for NPs production (e and f, resp.) were used to confirm their similar differentiation capacity (original magnifications: 100x).

Following 10 days of culture under differentiation conditions, cells were stained by Alizarin Red and Oil Red O to confirm calcium deposition and accumulation of lipid vacuoles in cytoplasm of the MSCs, respectively. Microscopic examinations revealed no remarkable differences between osteogenic differentiation capacities of unlabeled MSCs (Figure 2(a)) and DiD-labeled MSCs (Figure 2(b)). Likewise, DiD-labeled MSCs (Figure 2(d)) showed the same adipogenic differentiation capacity as the intact MSCs (Figure 2(c)). In addition, differentiation of unlabeled and DiD-labeled MSCs into NPs was also assessed after seven days of culture in proper differentiation media. Microscopic examination demonstrated the differentiation of untagged MSCs (Figure 2(e)) as well as DiD-labeled MSCs (Figure 2(f)) into NPs according to neurosphere formation. In addition, while mRNA expression level of nestin was increased almost 2-fold in NPs compared to MSCs, no significant difference was observed in nestin mRNA expression between NPs derived from unlabeled MSCs and DiD-labeled MSCs (2.3 ± 0.4 and 2.16 ± 0.2, resp.; *P* = 0.7).

### 3.3. Mitochondrial Membrane Potential and ROS Production in DiD-Labeled MSCs

**Figure 3:**
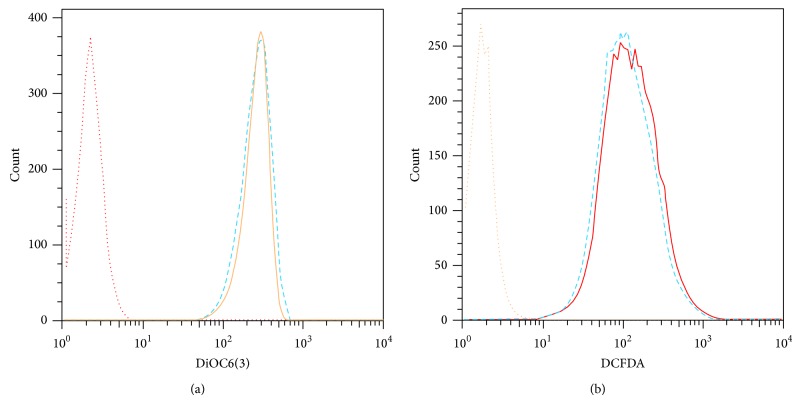
Flow cytometric analysis of ROS production and mitochondrial membrane potential of MSCs. (a) DiD-labeled MSCs stained with DiOC6(3) (dash line) emitted quite similar fluorescent intensity compared to unlabeled MSCs (solid line) at 520 nm. (b) No significant differences in fluorescent intensity of DCFDA were also observed between DiD-labeled (dash line) and unlabeled MSCs (solid line). These data were expressed as mean ± SD of three independent experiments in triplicate.

In the context of toxicity of DiD labeling, mitochondrial membrane potential and ROS production by DiD-labeled MSCs were assessed by DiOC6(3) and DCFDA labeling, respectively. The result showed that there were no significant differences in mean fluorescent intensity (MFI) of DiOC6(3) between DiD-labeled and unlabeled MSCs (259 ± 14.2 and 239.6 ± 15.3, resp.; *P* = 0.35; Figure 3(a)). To further consolidate safety of DiD labeling on MSCs function, the possible occurrence of apoptosis was also investigated by DCFDA staining. No significant differences in MFI of DCFDA were observed between DiD-labeled and unlabeled MSCs (157 ± 15.68 and 149 ± 20.9, resp.; *P* = 0.52; Figure 3(b)). Therefore, the results confirmed DiD labeling did not affect the viability of MSCs.

### 3.4. PGE2 and IL-10 Production by DiD-Labeled MSCs

The results of the present study showed that unlabeled and DiD-labeled MSCs are able to produce detectable and statistically similar levels of PGE2 (5316 ± 328 and 5338 ± 109 pg/mL, resp.; *P* = 0.91; Figure 1(b)) and IL-10 (21.0 ± 2.3 and 18.53 ± 5.5 pg/mL, resp.; *P* = 0.5; Figure 1(c)) after 24 hours of culture. There were also no significant statistical differences in PGE2 production (6058 ± 424 and 5828 ± 229 pg/mL, resp.; *P* = 0.45) or IL-10 synthesis (23.5 ± 2.3 and 27 ± 5.7 pg/mL, resp.; *P* = 0.2) between unlabeled and DiD-labeled MSCs subsequent to 48 hours of culture.

### 3.5. Inhibition of Splenocyte's Proliferation and Cytokine Production by DiD-Labeled MSCs

**Figure 4:**
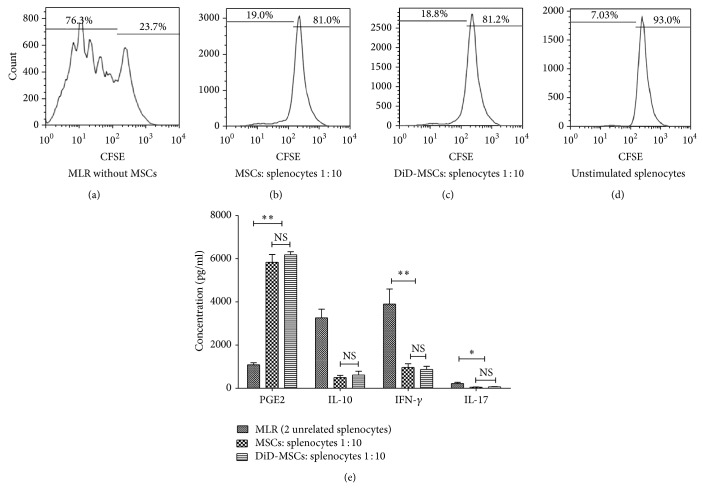
MSCs inhibit proliferation and cytokine production by activated splenocytes. Unlabeled and DiD-labeled BALB/c-derived MSCs were added into 2-way MLR reaction of splenocytes (at ratio 1 : 10) and the proliferation of splenocytes (based on CFSE reduction) was checked after 5 days. Histogram of cell proliferation in MLR reaction in the absence of MSCs (a) and in the presence of unlabeled (b) or DiD-labeled MSCs (c) demonstrated suppression of splenocytes proliferation by MSCs, whether labeled with DiD or not. Unstimulated splenocytes showed the lowest level of CFSE dilution (d). Three days after initiation of MLR reaction, downregulation of IL-17, IFN-*γ*, and IL-10 and upregulation of PGE2 were observed in the culture supernatants of MLR reactions in the presence of unlabeled or DiD-labeled MSCs (e). NS: not significant; ^*^ *P* < 0.05; ^**^ *P* < 0.01. These data were expressed as mean ± SD of three independent experiments in duplicate.

To test the antiproliferative function of MSCs on splenocytes, MLR reaction was conducted in the presence and absence of MSCs. Evident proliferation was detected in two-way MLR in the absence of MSCs (71.15 ± 7.25%; Figure 4(a)), while the proliferative response was significantly decreased in 1 : 10 cell ratio of MSC/splenocyte, whether MSCs were unlabeled (21.42 ± 7.66%, *P* = 0.0001; Figure 4(b)) or labeled with DiD (22.78 ± 5.90%, *P* = 0.0001; Figure 4(c)). This observation could be a result of PGE2 overproduction since analysis of MLR supernatants revealed upregulation of PGE2 in the presence of unlabeled and DiD-labeled MSCs (5832 ± 362 pg/mL and 6181 ± 147 pg/mL, resp.) in comparison to supernatant of MLR reaction in the absence of MSCs (1089 ± 93.8 pg/mL; *P* = 0.05 for both comparison; Figure 4(d)). Interestingly, as shown in Figure 4(d), in comparison to MLR in the absence of MSCs, the presence of unlabeled and DiD-labeled MSCs significantly decreased the levels of IL-10 (3260 ± 400 pg/mL, 500 ± 96 pg/mL, and 618 ± 174 pg/mL, resp.; *P* = 0.05 for both), IFN-*γ* (3903 ± 697 pg/mL, 963 ± 168 pg/mL, and 873 ± 147 pg/mL, resp.; *P* = 0.05 for both), and IL-17 (223 ± 52.6 pg/mL, 51.5 ± 10.4 pg/mL, and 67.6 ± 7.3 pg/mL, resp.; *P* = 0.05 for both) in the culture supernatant while the levels of the abovementioned cytokines were not significantly different between supernatants collected from MLR in the presence of unlabeled and labeled-MSCs.

### 3.6. Improvement of Clinical Score of EAE

**Figure 5:**
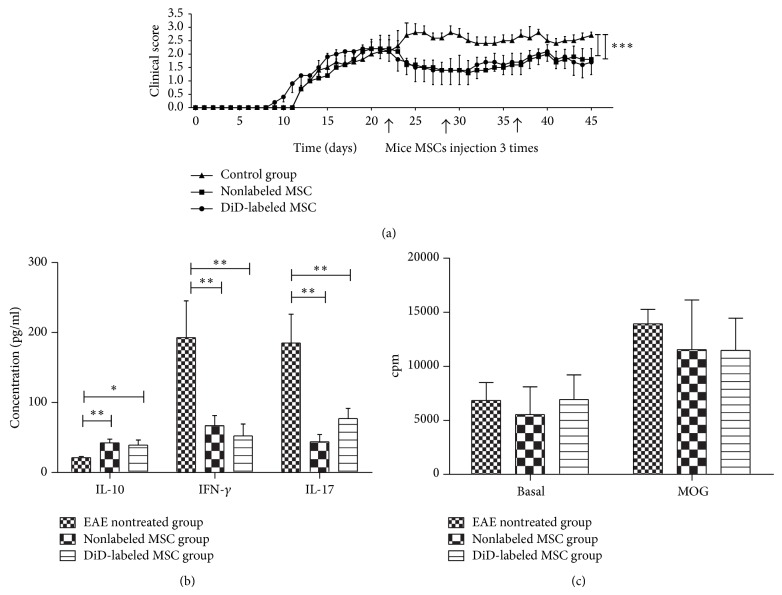
Therapeutic effects of allogenic MSCs in EAE mice. The mean ± SEM of clinical score of EAE in mice treated with three intraperitoneal injections of 1 × 10^6^ allogeneic MSCs with or without DiD as well as EAE controls is depicted in (a). MSCs with or without DiD labeling decrease the levels of IFN-*γ* and IL-17 and increase IL-10 levels in sera of treated mice compared to those in control EAE group (b).* In vitro* stimulation of splenocytes by MOG_35–55_ peptide revealed no significant reduction in splenocytes proliferation in treatment groups compared to those of control group (c). ^*^ *P* < 0.05, ^**^ *P* < 0.01, and ^***^ *P* < 0.001. These data were collected as mean ± SD from five mice in each group.

Three times injection of allogeneic MSCs with or without DiD labeling were similarly reduced clinical score of EAE (1.65 ± 0.24 and 1.67 ± 0.21, resp.) compared to those of control untreated-EAE mice (2.56 ± 0.17; *P* = 0.001 for both comparisons; Figure 5(a)). In agreement with clinical scores, sera levels of proinflammatory cytokines (IFN-*γ* and IL-17) were decreased significantly in the sera of mice treated with labeled (52.0 ± 37.1 pg/mL and 77.0 ± 31.1 pg/mL, resp.; *P* = 0.008 and *P* = 0.01, resp.; Figure 5) or unlabeled MSCs (67.0 ± 32.2 pg/mL and 43.0 ± 29.9 pg/mL, resp.; *P* = 0.01 and *P* = 0.007, resp.; Figure 5) compared to those in untreated control mice (143.7 ± 117.0 pg/mL and 185.0 ± 91.6 pg/mL, resp.). Of interest, the levels of IL-10 were significantly higher in untreated control mice (21.0 ± 3.8 pg/mL) compared to those in mice treated with labeled MSCs (39.0 ± 15.9, *P* = 0.01; Figure 5(b)) or unlabeled MSCs (42.0 ± 12.1, *P* = 0.008; Figure 5(b)). Similar to proinflammatory cytokines, no significant difference in the levels of IL-10 was observed between mice treated with unlabeled MSCs and DiD-labeled MSCs (42.4 ± 12.1 pg/mL and 39.0 ± 15.9 pg/mL, resp.; *P* = 0.7; Figure 5(b)). Also,* in vitro* stimulation of splenocytes from EAE-treated mice with MOG_35–55_ peptide showed lesser proliferation than control untreated mice, though this difference was not significant (*P* = 0.13 for unlabeled and *P* = 0.2 for DiD-labeled MSCs group, resp.; Figure 5(c)).

## 4. Discussion

Recent studies have shed light on the MSCs as potent candidates for treatment of inflammatory and autoimmune-mediated diseases. Privileged advantages of MSCs include their ability in differentiation to various cell types [1, 2, 20] and suppressive impact on immune responses [3, 21]. However, lack of distinctive and authenticated markers on MSCs for* in vivo* tracing has made the researchers employ various nonspecific tracking methods. Tracking of MSCs with optical imaging using biocompatible fluorescent dyes such as DiI has been assessed in several studies [22, 23]. However, usage of “DiD” with long wavelength emission spectra is preferred due to the lower interference with background fluorescence [10, 11]. Accordingly, DiD can be considered as a suitable substitute for DiI. Due to the lack of published investigations on possible effects of DiD labeling on biological functions of MSCs, in this study the consequences of DiD labeling on viability, proliferation, immunosuppressive activity, and differentiation potential of MSCs were investigated. The results of the present study have shown that labeling with 5 *μ*M DiD did not induce the production of ROS in MSCs. Furthermore, as a marker of early apoptosis, mitochondrial membrane potential in the DiD-labeled MSCs remained unchanged. Furthermore, no significant differences were observed between the DiD-labeled and control MSCs regarding their proliferative potency. As a result, 5 *μ*M DiD have neither apoptotic nor cytostatic/cytotoxic effects on MSCs. This result is compatible with previous reports indicating labeling with 4 *μ*M DiI has no cytostatic/cytotoxic effects on MSCs [22, 23].

The undesirable effect of labeling on the differentiation capacity of MSCs has always been another apprehension of researchers. Current study showed that DiD labeling does not affect differentiation capability of MSCs to different progenitors including adipocytes and osteocytes and differentiation towards neural precursors at the morphological and molecular levels (expression of nestin mRNA). Sutton et al. showed that DiD-labeled human MSCs maintained their capability to differentiate into chondrocytes lineage [10]. Although the glycosaminoglycan level of the labeled MSCs was reduced upon differentiation, labeling had no evident effects on cell morphology [10]. Weir et al. [22] and Dai et al. [24] have also shown that labeling of MSCs with DiI did not interfere with their capacity to differentiate into cardiomyocytes. Overall, in contrast to some reports that have revealed the negative effects of the* CdSe/ZnS* quantum dot labels or Feridex tracers on the MSCs' differentiation capacity [25, 26], DiD had no effects on the mice MSCs differentiation.

It has been shown that MSCs can inhibit immune cells functions by either physical contact or secretion of soluble factors [27–30]. Therefore, we investigated whether labeling of MSCs with 5 *μ*M DiD might affect the immunosuppressive ability of MSCS in prevention of splenocytes proliferation and cytokine production in MLR assay. The results of the present study showed that unlabeled and DiD-labeled MSCs have the same ability in production of two well-known inhibitory mediators (PGE2 and IL-10; Figure 3) that inhibit lymphocytes activation [31, 32]. In accordance with these results, DiD-labeled MSCs were able to decrease the proliferation of splenocytes in two-way MLR as efficiently as the unlabeled MSCs (*P* = 0.001; Figure 4). In addition, the production of inflammatory (IFN-*γ* and IL-17) and anti-inflammatory (IL-10) cytokines in the culture supernatant of MLR was significantly reduced in the presence of both DiD-labeled and unlabeled MSCs (Figure 4(d)). It can be hypothesized that production of high levels of PGE2 by both DiD-labeled and unlabeled MSCs (Figure 3(b)) might lead to prevention of lymphocyte activation and cytokine expression. In this respect, inhibitory effect of PGE2 on IFN-*γ* production [33] or inhibition of lymphocyte proliferation by MSCs through PGE2 production [34] has been reported previously. The effects of PGE2 on IL-10 production are controversial [35–38]. However, given the fact that PGE2 is involved in suppression of IL-10 production in certain conditions [37, 38], in our results reduced production of IL-10 in the presence of DiD-labeled or unlabeled MSCs might be explained according to the presence of high levels of PGE2 in the coculture of MSCs and splenocyte [37, 38]. Overall, both intact and DiD-labeled MSCs are equally competent in production of PGE2 and employment of other mechanisms that eventually reduce cytokines production by splenocytes in MLR assay.

The present study indicated that, similar to unlabeled MSCs, DiD-labeled MSCs significantly improve the clinical scores of the EAE compared to the untreated controls (1.67 ± 0.21 and 1.65 ± 0.24 versus 2.56 ± 0.17, resp.; *P* = 0.0001). In addition, in comparison to untreated controls, DiD-labeled and unlabeled MSCs-treated mice showed significant reduction in the serum levels of IFN-*γ* (193 ± 117 pg/mL versus 52 ± 37.1 pg/mL and 67 ± 32.2 pg/mL, resp.; *P* = 0.01) and IL-17 (185 ± 91 pg/mL versus 77 ± 31.1 pg/mL and 43 ± 29.9 pg/mL, resp.; *P* = 0.01) and increased levels of IL-10 (21.0 ± 3.8 pg/mL versus 39.0 ± 15.9 pg/mL and 42 ± 12.1 pg/mL, resp.; *P* = 0.05 and *P* = 0.01, resp.). These results can be explained by improved peripheral tolerance resulting from DiD-labeled or unlabeled MSCs administration and deviation of cytokine profile to Th2/Treg pattern. As a result, our study revealed no significant differences between the* in vivo* effects of unlabeled and DiD-labeled MSCs in the reduction of the disease symptoms. However, the specificity of DiD labeling in tracing of MSCs needs to be investigated in more detail since the possibility of DiI dissociation from labeled cells and donor-to-host transfer of dye [39] has been reported.

## 5. Conclusion

The results of the present study indicated that DiD might be an appropriate candidate dye for MSCs labeling and* in vivo* tracking since DiD labeling did not show any effect on different biological behaviors and functions of the mice MSCs in both* in vitro* and* in vivo* conditions.
